# mRNA-Seq of Single Prostate Cancer Circulating Tumor Cells Reveals Recapitulation of Gene Expression and Pathways Found in Prostate Cancer

**DOI:** 10.1371/journal.pone.0049144

**Published:** 2012-11-07

**Authors:** Gordon M. Cann, Zulfiqar G. Gulzar, Samantha Cooper, Robin Li, Shujun Luo, Mai Tat, Sarah Stuart, Gary Schroth, Sandhya Srinivas, Mostafa Ronaghi, James D. Brooks, AmirAli H. Talasaz

**Affiliations:** 1 Department of Diagnostic Research, Illumina, Inc., Hayward, California, United States of America; 2 Department of Urology, Stanford University Medical Center, Stanford, California, United States of America; 3 Department of Medicine, Division of Oncology, Stanford University Medical Center, Stanford, California, United States of America; University of Kentucky College of Medicine, United States of America

## Abstract

Circulating tumor cells (CTC) mediate metastatic spread of many solid tumors and enumeration of CTCs is currently used as a prognostic indicator of survival in metastatic prostate cancer patients. Some evidence suggests that it is possible to derive additional information about tumors from expression analysis of CTCs, but the technical difficulty of isolating and analyzing individual CTCs has limited progress in this area. To assess the ability of a new generation of MagSweeper to isolate intact CTCs for downstream analysis, we performed mRNA-Seq on single CTCs isolated from the blood of patients with metastatic prostate cancer and on single prostate cancer cell line LNCaP cells spiked into the blood of healthy donors. We found that the MagSweeper effectively isolated CTCs with a capture efficiency that matched the CellSearch platform. However, unlike CellSearch, the MagSweeper facilitates isolation of individual live CTCs without contaminating leukocytes. Importantly, mRNA-Seq analysis showed that the MagSweeper isolation process did not have a discernible impact on the transcriptional profile of single LNCaPs isolated from spiked human blood, suggesting that any perturbations caused by the MagSweeper process on the transcriptional signature of isolated cells are modest. Although the RNA from patient CTCs showed signs of significant degradation, consistent with reports of short half-lives and apoptosis amongst CTCs, transcriptional signatures of prostate tissue and of cancer were readily detectable with single CTC mRNA-Seq. These results demonstrate that the MagSweeper provides access to intact CTCs and that these CTCs can potentially supply clinically relevant information.

## Introduction

Circulating tumor cells (CTC) are cells that part from a primary tumor or metastasis and enter the blood stream via the leaky vasculature that arises around a growing tumor. Once in the blood, CTCs encounter damaging stresses associated with hemodynamic shear, low oxygen conditions, lack of anchorage sites, and immune system attack [1], [2]. A small number of CTCs survive however and extravasate into surrounding tissues to seed metastasis or reseed the primary tumor [3]. Described over a century ago [4], CTCs can be now enumerated using the FDA approved CellSearch platform to provide prognostic information regarding survival for metastatic breast, colon and prostate cancer patients [5]–[7]. Moving beyond enumeration, several groups have suggested that genetic and transcriptional analysis of individual CTCs might be leveraged to make personalized medical decisions for cancer therapy and provide insights into the biological processes involved in metastasis [8]–[10].

Several methods have been exploited to isolate CTCs from red and white blood cells (WBCs). Differentiating physical properties and surface markers of CTCs have been utilized for their isolation by filtration [11], microfluidic chip [12], [13], buoyant density centrifugation [14], immunomagnetic selection [15], [16], functional enrichment and detection [17], [18], and automated immune microscopy [19], [20]. Immunomagnetic enrichment with anti-EpCAM beads followed by fluorescence activated cell sorting has recently been shown to be an effective approach for isolating CTCs relatively free of hematopoietic cells [21]. Of the platforms currently in use for isolating CTCs, the MagSweeper technology provides great ease of use and access to highly pure, intact, individual CTCs suitable for genetic and proteomic analysis [22], [23].

CTCs are generally present in low numbers in patient blood samples (typically 1 per 10^7^ nucleated cells in blood) so extracting maximal information from single or available CTCs isolated from a patient's blood sample is essential. Next generation DNA sequencing is particularly well suited for deep interrogation of cancer genomes and transcriptomes [24] even when applied at the single cell level [25]. In this study, we validated the performance of a new generation of the MagSweeper using spiked LNCaP prostate cancer cells in normal blood. We then conducted a capture sensitivity comparison of prostate cancer CTCs between CellSearch and the MagSweeper on replicate patient samples. Whole transcriptome sequencing studies of single LNCaP cells revealed that MagSweeper isolation has minimal effects on gene expression. Furthermore, mRNA-Seq mediated transcriptome profiles of individual prostate CTCs isolated from metastatic patient blood were compared to normal prostate tissue samples and single prostate cancer cell lines. Despite cell to cell heterogeneity and a wide range of CTC RNA quality, higher expression of prostate related genes such as the androgen receptor (AR), KLK3 (PSA) and TMPRSS2 could be distinguished in prostate CTCs. Bioinformatic screens for genes expressed 100 fold higher in CTCs compared with normal prostate samples revealed other known gene pathways and signatures expected of prostate cancer and their host's treatment history.

## Materials and Methods

### Ethics Statement

This study was reviewed and approved by Stanford's Human Subjects Research Compliance Board and adhered to HIPPA regulations. All human subjects signed informed consent prior to blood sample collection.

### Patient samples and blood collection

Patient samples were collected in 10 ml EDTA tubes (Beckton Dickenson) and processed within 12 hours of collection. Samples were collected according to guidelines specified and approved by an Institutional Review Board and after informed consent. For comparisons between MagSweeper and CellSearch, a second 7.5 ml blood sample was collected in a CellSave tube. CellSearch assays were performed by Quest diagnostics. Total RNA from three histologically normal prostate tissues were obtained from surgically removed prostates under a separate IRB-approved protocol.

### Cell Culture and Cell Spiking

LNCaP, PC-3, and T24 cells were purchased and cultured according to conditions specified by the American Type Culture Collection (ATCC). Following dissociation live and dead cells were determined by Trypan blue exclusion. For spiking, cells were diluted to approximately 3×10^3^ cells per ml and cell concentration verified by spotting and counting six, ten microliter aliquots of cells spotted on a glass microscope slide. No correction for dead cells based on Trypan blue exclusion was used prior to spiking cells.

### Bead Binding and Cell Surface Staining

Custom 1.5 um Streptavidin coated magnetic beads were functionalized with a custom biotinylated monoclonal antibody directed against extracellular human EpCAM epitope. Two 3.75 ml volumes of blood per sample were subjected to red blood cell lysis with 10 volumes of 1× PharmLyse (BD Biosciences) for 15 minutes at room temperature. Remaining cells were pelleted at 4°C for 15 min at 300× g. Cell pellets were transferred with 2×1 ml aliquots of 1%BSA/PBS/5 mM EDTA into a 2 ml flat bottom microcentrifuge tube (VWR International). Cells were then pelleted for 5 minutes at 510× g. Cell pellets were resuspended in a total volume of 1 ml of 1%BSA/PBS/5 mM EDTA containing 15 ul of Alexa 488 anti-human CD45 (Life Technologies) and 30 ul of our custom anti-EpCAM beads. Samples were rotated for 30 minutes at 4°C followed by addition of 20 ul of Phycoerythrin (PE) anti-human EpCAM monoclonal antibody (BD Biosciences 347198). Samples were then rotated at 4°C for an additional 30 minutes and then transferred to a well in a 6 well plate containing 6 ml of 1%BSA/PBS/5 mM EDTA. Samples were mixed once by pipetting up and down in a 10 ml pipette, and plates were spun for 5 minutes at 400 rpm, followed by incubation for 15 minutes at 4°C prior to MagSweeper isolation. In some spiking experiments, LNCaP cells were labeled prior to spiking with CFDA (Life Technologies) following manufacturer's instructions.

### MagSweeper and Single Cell Isolation

Putative CTCs were isolated using two rounds of MagSweeper isolation. Cells isolated after the first round of MagSweeping were released and stained in a well of a 6-well plate containing 500 nM membrane impermeable DAPI (Life Technologies) so that membrane compromised cells could be identified. Following a second round of MagSweeping cells were dispersed and pelleted at 400 rpm for 1 min in a well of a 6-well low adhesion plate in 10% Superblock (ThermoFisher)/PBS. Wells were viewed with an Olympus inverted microscope equipped for epiflouresence. Putative CTCs were identified as cells that stained positive for PE anti-EpCAM and negative for Alexa 488 anti-CD45. Putative CTCs that were DAPI+ were excluded from further analysis. DAPI negative putative CTCs were isolated in 1 ul of 10% Superblock/PBS with a pipetteman into a 0.2 ml PCR tube containing 2.5 ul of 5% Ribonuclease Inhibitor (Life Technologies)/0.2% Triton X-100 (10% solution, Sigma) prepared in nuclease free water. Collected cells were flash frozen on dry ice and stored at −80°C. Cell purity was measured as the number of spiked cells recovered divided by the number of spiked cells recovered plus the number of leukocytes.

### Single Cell mRNA-Seq

Single cells were lysed and RNA reverse transcribed using the SMARTer Ultra Low Input RNA for Illumina Sequencing kit (Clontech). cDNA was amplified using the Advantage 2 PCR kit (Clontech) for 18–25 cycles prior to conversion into a Illumina compatible DNA sequencing library using the Nextera DNA Sample Prep Kit (Illumina) and 12 cycles of PCR to amplify the library. Libraries were quantified using a BioAnalyzer (Agilent) and qPCR using a Kappa Syber Green PCR kit (Kappa Biosciences) on an IIlumina ECO qPCR machine. Paired end flow cells were prepared using 8 pM of Nextera library per lane on a cBot (Illumina) and sequenced using single 50 bp reads on an Illumina GAIIx .

### Alignment

Sequencing data was collected with RTA version 1.9 and fastq files were generated with Casava 1.8. Reads that did not pass Illumina's standard quality filter were removed by default. Reads were aligned to hg19 with tophat v1.3.3 and counts per transcript were calculated for hg19 in iGenomes using cufflinks v1.1 with the options: –GTF <genes.gtf> –max-bundle-frags 20000000. Gene-by-gene raw counts and fragments per kilobase of exon per million fragments mapped (FPKM) were generated from the cufflinks isoform.fpkm_tracking file by identifying all the transcripts with the same gene name and taking, respectively, the sum of coverage multiplied by transcript length/read length for each transcript and the sum of the FPKM for each transcript. The raw counts and FPKMs were used in all the downstream analysis, except the QC step.

### RNA-Seq Data Quality Control

Quality control (QC) was done using an internal Illumina RNA-Seq QC script. Base-by-base coverage was calculated across a hand-picked set of 600 quality control genes that are chosen from RefSeq and are highly mappable and highly abundant in universal human RNA (UHR) samples (Agilent) . Highly mappable means that >90% of bases for a selected transcript have 100% mappability. High expression in UHR means that only genes with average coverage >1× were selected. So for a given transcript 1000 bp in length there are at least 20 reads of 50 bp mapped to it. Genes in this set with greater than 1 FPKM were used to calculate additional statistics. The median coverage across all the length-normalized QC genes with greater than 1 FPKM was plotted. For each quality control gene with greater than 1 FPKM, the coefficient of variation was calculated across the gene, and the median of all the CVs was determined.

### Unsupervised Clustering

Unsupervised hierarchical clustering [26] was done with the heatmap function in R, with the default Euclidean distance used as the dissimilarity metric. For each sample, the 100 genes with the highest FPKM were selected and the resulting pool of 312 genes was used in the clustering.

### LNCaP expression analysis

EdgeR [27] was run using moderated tagwise dispersions with the raw counts per gene as input. Correlation between samples was calculated based on the raw number of reads mapped to each gene.

### Genes over-expressed in CTCs

Due to the inherent variability in single cell data, coupled with the varying degrees of apparent mRNA decay observed in the CTCs, a simple thresholding method was used to identify over-expressed genes. For each gene the ratio of the 2^nd^ highest FPKM value among the set of CTCs to the FPKM in the normal prostate RNA was calculated. Genes with a ratio of at least 100× and at least 10 total reads in one of the CTCs were selected. GO ontologies were generated using the Panther Classification System (http://www.pantherdb.org/) [28].

## Results

### MagSweeper metrics for CTC isolation

**Figure 1:**
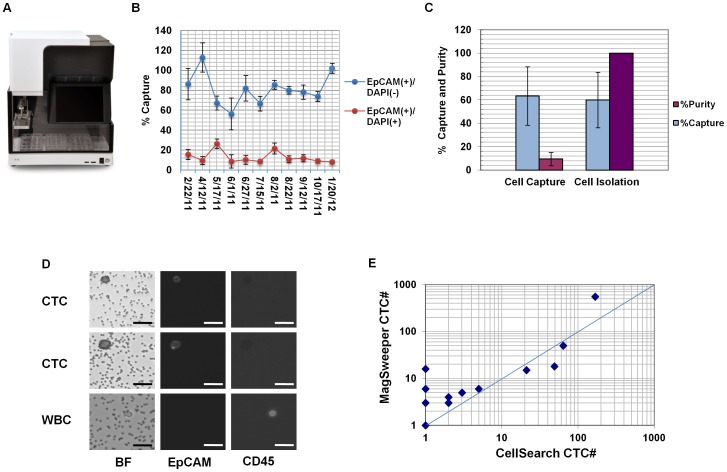
Metrics of MagSweeper circulating tumor cell (CTC) isolation. (A) Image of hood prototype of the MagSweeper. (B) Percent capture of 100 LNCaP cells spiked into 3.75 ml of normal blood (N = 54 experiments and 11 donors). Blue circles show mean percent recoveries of live EpCAM (+)/CD45 (−)/DAPI(−) cells and red circles show mean recoveries of membrane compromised EpCAM (+)/CD45 (−)/DAPI(+) cells. Error bars represent +/−1 S.D. (C) Percentage capture and purity of 10 LNCaP cells isolated following spiking into 7.5 ml of normal blood. Blue bars are the mean percent recovery of cells after MagSweeper isolation (Cell Capture) and pick and manual place single cell isolation (Cell Isolation) while purple bars show purity of LNCaP cells after MagSweeper and single cell isolation (N = 6 experiments and 4 donors). Cell purity was calculated as the number of spiked cells isolated divided by the number of spiked cells counted plus white blood cells counted. Error bars represent +/−1 S.D. (D) Bright field (BF) and images of fluorescently stained CTCs isolated from a prostate cancer patient blood sample, and contaminating WBC found after MagSweeper isolation. Scale bar = 20 microns. (E) MagSweeper versus CellSearch comparison of patient samples. Samples with 0 CTC were assigned a value of 1 for plotting purposes.

A new prototype of the Magsweeper [22] was developed that is compatible with operation in most biosafety cabinets (Figure 1A). To assess the performance of this platform, over an 11 month period, 100 LNCaP cells were repeatedly spiked into 3.75 ml of normal blood and isolated with the MagSweeper (n = 54 experiments and 11 blood donors). Prior to spiking, LNCaP cell viability measured by Trypan blue exclusion was 89%±8%. During isolation from spiked blood LNCaP cells were fluorescently labeled with antibodies against EpCAM and CD45 to distinguish LNCaP cells from WBCs, and the membrane impermeable nuclear stain DAPI was used to identify dead (membrane compromised) cells. Post-isolation we recovered a mean of 81%±16% of live spiked LNCaP cells (EpCAM+/CD45−/DAPI−) (Figure 1B, blue line) while DAPI staining revealed an additional 12%±6% of the isolated LNCaP cells that were membrane compromised (EpCAM+/CD45−/DAPI+). Since the original cell spike contained 11% dead cells (measured by Trypan blue exclusion) recovery of 12% DAPI positive cells following MagSweeping indicates that damage to spiked LNCaP cells during the entire procedure is minimal. Normal, unspiked blood samples failed to yield EpCAM positive cells (data not shown).

To assess purity of ultra-rare cells after MagSweeping, 7.5 ml of normal donor blood was spiked with 10 CFDA labeled cells (n = 6 experiments and 4 donors) and processed according to our protocol for CTC isolation. Captured LNCaP cells were identified by fluorescent detection of CFDA, while contaminating WBCs were identified by positive CD45 staining. Following magnetic sorting a mean percentage LNCaP recovery of 63%±25% was observed. Enumeration of CD45 positive cells yielded an initial purity of isolated LNCaP cells after MagSweeper cell isolation of 10%±6% and 100% post single-cell isolation using a pick and place method (Fig. 1C).

We next performed a comparative analysis of MagSweeper versus CellSearch in enumeration of CTC in blood samples from patients with metastatic prostate cancer. At the time of draw, two 7.5 ml samples of blood were collected, one sample in a standard EDTA tube for MagSweeper cell isolation and a second in a CellSave tube for analysis by an independent lab (Quest Diagnostics) using the CellSearch assay. Immunofluorescent staining of MagSweeper isolated cells identified CTCs as EpCAM positive and CD45 negative. CTCs were easily distinguished from WBC which were CD45 positive, EpCAM negative (Fig. 1D). Numbers of completely purified CTCs enumerated by MagSweeper isolation and CellSearch were compared and found to be reasonably similar, with a mild trend observed toward better CTC capture using the MagSweeper in samples with low CTC numbers (Figure 1E).

### MagSweeper isolation has minimal effects on single cell transcriptomes

**Figure 2:**
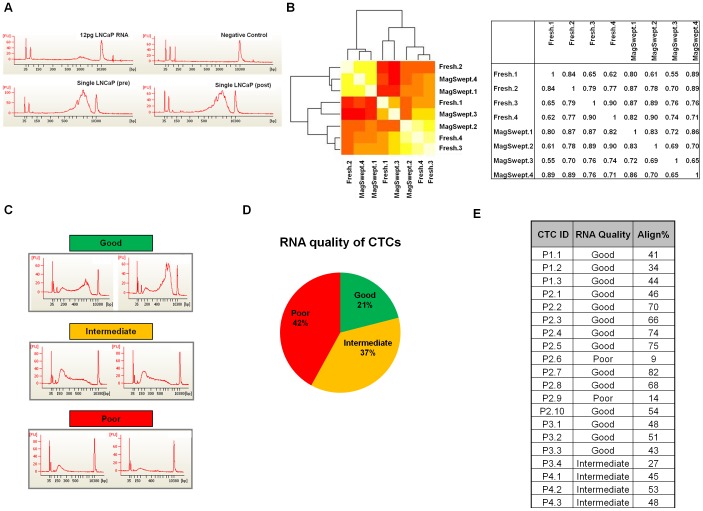
MagSweeper isolation has minimal effects on single cell transcriptomes. (A) Bioanalyzer traces of amplified cDNAs from single LNCaP cells pre (Single LNCaP(pre)) and post MagSweeping (Single LNCaP (post)), and positive control (12 pg of LNCaP total RNA) and negative control (Negative control). (B) Heatmap of correlations between fresh and MagSwept single-cell RNA-Seq data and table of correlations between fresh and MagSwept samples. Yellow indicates higher correlations and red lower correlations. (C) Representative bioanalyzer traces of good, intermediate, and poor CTC cDNA amplification products. (D) Percent breakout of CTC RNA quality based on classification of cDNA amplification products – green indicates good quality, yellow samples are partially degraded RNA and red indicates degraded RNA samples. (E) Sequenced CTCs, their RNA quality and % alignment of passing filter mRNA-Seq reads to the human genome build hg19. Patient CTC ID indicates single patient CTCs identified as patient number. CTC number (P1.1). RNA Quality is based on bioanalyzer traces of amplified cDNA and Align% is alignment % of mRNA-Seq reads.

To assess whether the MagSweeper isolation process affected the global transcriptional profile of isolated cells, we performed single cell mRNA-Seq on 4 fresh LNCaP cells just prior to spiking into blood, and on 4 LNCaPs after MagSweeper isolation from spiked normal blood. Isolated cells were stored frozen for at least a month to simulate storage conditions. BioAnalyzer traces of amplified cDNA from a fresh and a MagSweeper isolated cell revealed similar molecular weight peaks of amplification products centered at approximately 1000 base pairs (Fig. 2A).

To study genes differentially expressed between fresh and MagSweeper isolated single cells, we first used the Bioconductor edgeR package. We identified only 1 gene as differentially expressed between MagSweeper-isolated and control LNCaP cells at a false discovery rate (FDR) cutoff of 0.05 and none at a cutoff of 0.01.

We also explored several other methods for identifying differences between the fresh and MagSweeper isolated cells. First, to characterize the degree of cell-to-cell variability, we asked how many genes had a very high FPKM (>10) in at least 1 sample and a very low FPKM (<0.1) in at least one sample. We considered three different groups of samples: (1) 4 fresh cells (2) 4 MagSwept cells and (3) 2 fresh and 2 MagSwept cells. The number of highly variable genes in four fresh cells (215), four MagSwept cells (268) and a combination of 2 fresh and 2 MagSwept cells (239) was similar in all groups and likely reflects cell to cell heterogeneity. In another comparison we calculated the correlation of the number of reads per gene between each pair of samples, and found that the within-group correlations were no stronger than the between group correlations – that is, the cells did not cluster based on isolation method (Figure 2B). Finally in a separate study, we ran an Illumina expression microarray on pools of 10,000 LNCaP cells pre- and post-MagSweeper isolation. Again, there was high cross-correlation (R^2^ = 0.985) between MagSweeper isolated cells and controls indicating that MagSweeper produces minimal alterations in gene expression (data not shown).

### mRNA-Seq of single prostate cancer CTCs

We prepared amplified cDNA from 67 CTCs isolated from 13 prostate cancer patients. CTCs were isolated after MagSweeping based on immunofluorescent staining for cells that were (EpCAM+/CD45−/DAPI−). Unlike cultured LNCaP cells (Figure 2A), we found that there was a wide range of sizes of amplified cDNAs in patient-derived CTCs, reflective of initial RNA quality. We characterized traces of amplification products into 3 groups: 1) those with peaks centered around 1000 bps (good quality), 2) traces with intermediate length amplification products (partially degraded), and 3) traces with predominantly low molecular weight amplification products (degraded) (Fig. 2C). Looking across all 67 CTCs, 21% had good quality RNA, 37% were partially degraded, and 42% of the samples were degraded (Fig. 2D). RNA quality tended to be somewhat patient specific - for example patient 2 yielded the highest number (8/12) of good quality RNA samples. Using RNA quality as a guide, we sequenced the libraries of 24 CTCs which included representatives of all three RNA quality classifications, and aligned sequences to the human genome (build hg19). Based on sequence alignment score of greater than five percent, sequence data for 20 CTCs collected from 4 patients (P1, P2, P3 and P4) were selected for further in depth study (Fig. 2E).

### mRNA-Seq data quality

**Figure 3:**
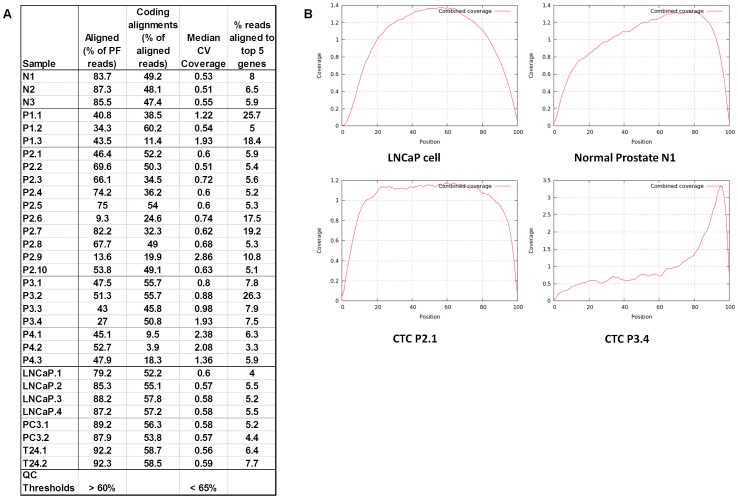
Alignment metrics of human prostate CTC mRNA-Seq sequences. (A) Percentage of passing filter (PF) reads that aligned, percentage of alignments that map to coding regions, median coverage CV, and percentage of reads that map to the five genes with the highest number of mapped reads. To calculate the “Median CV”, first the CV of coverage was calculated across each of 600 genes in a hand-picked set of quality control genes expressed in universal human RNA (Agilent), and then the median of these CVs is taken (considering only the genes with at least 1× coverage). The “% aligned” is the percentage of PF reads that align to the genome or to a splice site, excluding mitochondria and ribosomal RNA. Based on historical data, expected values for median CV and % aligned are <65% and >60%, respectively. Coding alignments are the % of reads that map to exons. The % of reads aligned to the top 5 genes for each sample are shown (B) Examples of the average length-normalized coverage across the 600 quality control genes, from samples LNCaP.3, N.1, P2.1, P3.4. Position 0 is the 5′-end of the transcripts and 100 is the extreme 3′-end of the transcript.

To assess the mRNA-Seq data quality, coverage was calculated using a quality control script and a handpicked set of 600 quality control genes that are highly mappable and expressed in universal human RNA (see Methods, RNA-Seq Data Quality Control for definition). The sequencing data from the single LNCaP, PC-3, and T24 cells passed the quality control standards for >60% alignment and <65% median alignment CV typically applied to large RNA input mRNA-Seq data sets (Fig. 3A). In contrast, CTCs displayed higher coverage median CVs and lower percentage of alignments than cultured cells. Typical coverage plots for cell lines, normal prostate tissues, and CTCs are shown in Figure 3B. The cell lines displayed smooth coverage across the length of the transcript, while the normal prostate samples had a slight 3′ bias, typical for tissue samples (Fig. 3B). The CTCs had a wide range of coverage bias, as shown. Since oligo-dT was used to prime cDNA synthesis, a 3-prime bias in the coverage data suggests mRNA degradation.

### mRNA-Seq data content

To understand transcript abundance, variation and range in CTCs, we compared the distributions of FPKM values of all RefSeq RNAs detected in CTCs and LNCaP cells (Tables S1 and S2). Measurements of RefSeq transcripts with ≥10 FPKM revealed in LNCaP cells (n = 4) 4622±136.2 transcripts (with a range of 4485 to 4786 transcripts). In contrast, in CTCs (n = 20) the number of RefSeq transcripts with ≥10 FPKM was 2362±865 transcripts (with a range of 1233 to 3987 transcripts).

**Figure 4:**
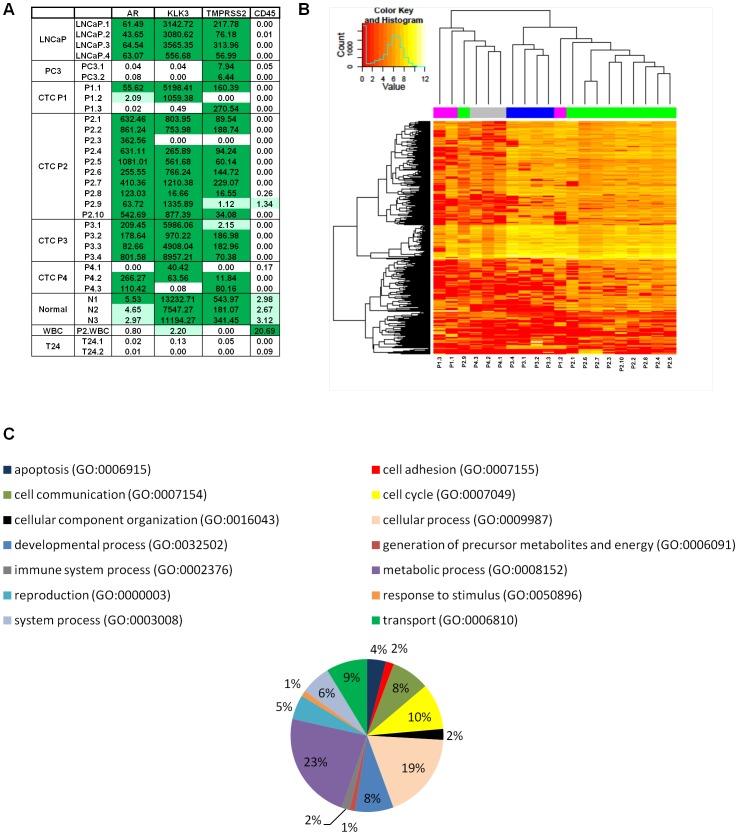
Expression, clustering, and functional classification of genes expressed in human prostate CTCs. (A) Expression of prostate cancer associated genes. For each gene, fragments per kilobase of exon per million fragments mapped (FPKM) for each CTC and controls are shown. FPKM values greater than 5 are shaded green and those with values between 1 and 5 are shaded light green. AR (androgen receptor), KLK3 (prostate specific antigen), and TMPRSS2are markers of prostate tissue. CD45 is a white blood cell marker. Prostate cancer cell lines include LNCaP and PC-3 while T24 is a bladder cancer, and WBC is a single white blood cell. Normal denotes normal prostate tissue. (B) Unsupervised clustering of over-expressed genes in patient CTCs. Colored bars across the top of the figure indicate different patients while individual patient CTCs are listed at the bottom of the cluster. (C) Functional classification of genes overexpressed in CTC using Gene Ontology (GO) classifications. For each functional grouping the % of genes over-expressed in each GO category is indicated.

Next, we looked at expression levels of several prostate makers and a leukocyte marker to establish that the patient-derived cells were of prostatic origin: androgen receptor (AR), prostate-specific antigen (PSA, KLK3), TMPRSS2, and the leukocyte marker CD45 in all CTC samples. Figure 4A shows the FPKM of each of these markers in patient CTCs, tissue culture cell lines and normal prostate, with values of >1 FPKM shaded in green. All but one of the CTCs was positive for at least one of the prostate markers. LNCaP and normal prostate showed expected expression of these prostate markers. PC-3 lacked KLK3 and AR expressed, as expected [29] but did express TMPRSS2. Importantly, all the CTCs and the cell lines were negative for the WBC marker CD45. Normal prostate, which is comprised of many cell types including WBCs, was positive for CD45 expression as was the single WBC that was subjected to mRNA-Seq. Finally, T24, a bladder cancer cell line did not express any of these markers. To understand how patient CTCs relate to one another, we performed an unsupervised clustering analysis of all patient CTCs. The analysis revealed that with the exception of two CTCs (P2.9 and P1.2), all CTCs from individual patients clustered in a patient specific manner (Fig. 4B).

### CTC Pathway Analysis

To find genes and pathways that were differentially activated in CTCs we compared transcript profiles of CTCs to those from normal prostate tissue and focused on genes that were over expressed in the CTCs. To normalize the RNA-Seq data based on gene sizes and number of mapped fragments, we used tophat and cufflinks to determine FPKM. We reasoned that the varying degrees of RNA degradation in the CTCs would lead to false-positive counting of under expressed genes in the CTCs, especially since the half-life of RNA varies from gene to gene. We used a manual thresholding method to identify genes that were overexpressed in CTCs. Specifically, we selected genes that were at least 100-fold higher in at least 2 of the CTCs compared to normal prostate tissue and that contained at least 10 mapped reads in at least one sample.

We identified 181 genes over-expressed in CTCs compared to normal prostate tissue (Table S3). To gain an overview of the range of biological functions associated with these transcripts, Gene Ontology annotations we derived from the GoSlim database using the Panther Classification System browser [28] (Fig. 4C) and categorized for biological processes. Out of 181 genes, 110 yielded 173 process hits which were classified into 14 biological processes. These were displayed using the Panther Pie Cart feature (Fig. 4C). Among the remaining 71 gene annotations not classified by GoSlim, 37 were non-coding RNAs including members of the MIR, SCARNA, SNAR, SNORA,SNORD and VTRNA families. The remaining 34 transcripts could be identified using GeneCards. Examination of the transcripts classified by biological processes revealed that one third were associated with either metabolic processes (23%, GO:0008152) or the cell cycle (10%, GO:0007049), consistent with mitotically active cells (Fig. 4C). Cell cycle and mitosis associated transcripts in the highly expressed gene set including TPX2, CCNA2, CCNB1 and B2, CDC20, CSK2, CDC2, CDKN3, CENPE, CD28 ,TOP2A, ORC1L, NUF2, CDK1, KIF2C, PTTG1 and TTK.

Interestingly, the list contains many transcripts germane to prostate cancer biology. For instance, all 3 CTCs from patient 1 expressed high levels of SPINK1, a transcript and protein identified as elevated in TMPRSS2-ERG fusion negative prostate cancers and associated with aggressive prostate cancer [30]. CTCs from patients 1 and 2 expressed high levels of BIRC5 (Survivin), an anti-apoptotic gene expressed at high levels in castration-resistant prostate cancer. CTCs also expressed cancer associated transcripts (BAGE, BAGE3, CT45A1, CT45A4, CT45A5, CT45A6, CTAG18, CTAG2, MAGEA12, MAGEA1, MAGEA3, MAGEA6, MAGEC1, MAGEC2, and PTTG1) and transcripts important in regulating development (HOXB7, HOXB8, HOXB9, NANOGNB, and LOC404266). Notably 4 transcripts (TOP2A, TK1, TPX2 and KIAA0101) expressed at high levels in CTCs were found in a list of 31 transcripts associated with disease recurrence after radical prostatectomy we published recently [31].

Using Ingenuity Pathway Analysis (IPA), we looked for overrepresented pathways and gene sets. The top *Diseases and Disorders* was Cancer and the top *Canonical Pathway* was “Cell Cycle: G2/M DNA Damage Checkpoint Regulation. Looking specifically at genes overlapping the IPA function *Prostate Cancer*, 9 genes were identified: AR, TK1, PLK1, MAGEA1, MAGEC1, MAGEC2, CTAGB1, BIRC5, and TOP2A. The CTCs from patient 2 contributed most significantly to these results. Repeating these analyses with only genes identified in P2 verses normal prostate tissue produced very similar results with highly significant p-values. Interestingly, excluding P2 from the analysis yields a weaker but still significant association with prostate cancer. This is consistent with the observation that the CTCs from P2 yielded significantly better quality libraries.

## Discussion

We have produced a new generation of MagSweeper which employs more sophisticated cell capture hardware and software than a previous version [22], and has a reduced footprint compatible with operation in most biosafety cabinets. These improvements combined with a multi-marker staining protocol allow the user to distinguish CTCs by fluorescent staining of cell surface markers. Validation of MagSweeper performance revealed that the mean capture of live LNCaP cells spiked into blood is 81%±16% which is comparable with the capture reported for high EpCAM expressing epithelial cancer cell lines spiked into blood on other CTC capture platforms [32]. In a comparative enumeration study with the CellSearch platform using prostate patient samples, MagSweeper allowed enumeration of comparable numbers of CTCs with a slightly better recovery of CTCs from patient samples with low starting numbers of CTCs (Fig. 1E). However, unlike other CTC isolation technologies (CellSearch cartridges, and CTC and OncoCEE chips) MagSweeper technology allows isolation and characterization of single CTCs, rather than pooled CTCs that are contaminated with variable numbers of WBCs (Fig. 1C, Cell Isolation, Fig. 4A). Furthermore, inclusion of DAPI as a dead cell exclusion marker allows discrimination of intact CTCs from damaged CTCs and CTC fragments that have been observed by several groups using the CellSearch [33], and other automated microscopy platforms [19], [34]. Although we have used EpCAM based capture and cell surface staining to isolate and identify single prostate cancer CTCs, combinations of capture and staining antibodies can be easily reconfigured for use with the MagSweeper to isolate CTCs from other malignancies or to isolate other cell types. Finally, single cell isolation using the MagSweeper does not appreciably alter gene expression. While single cells pre and post-MagSweeper isolation showed expected heterogeneity in transcriptome expression patterns from cell to cell, we were unable to find patterns of gene expression that were correlated with MagSweeper processing. This finding suggests that cell autonomous as opposed to extrinsic factors such as MagSweeper isolation govern gene expression at the level of the single cell (Fig. 2B).

The MagSweeper isolation protocol appears to be a relatively gentle method for isolating CTCs, with a mean cell attrition rate for spiked LNCaP of 1%. Furthermore, dead and membrane compromised cells are stained using DAPI allowing identification and isolation of live, membrane intact cells, and those cells most likely to yield intact DNA and RNA. With this protocol single LNCaP, PC-3 and T24 cells showed high quality RNA after amplification as judged by size of amplified cDNA (Fig. 2A) and passing mRNA-Seq quality control metrics for alignment, median alignment CV and positional coverage in a handpicked panel of 600 quality control genes (Fig. 3A and 3B). Therefore, we suspect that the heterogeneity in RNA quality present in CTCs isolated from patient samples (Fig. 2C, 3A and 3B) is due to features of CTC biology in vivo and not due to technical features of MagSweeper CTC isolation. Patients with the highest numbers of CTCs tended to yield CTCs with better RNA quality. Since all patients in this study were on therapy (Table S4), differences in RNA quality are likely related to treatment effects or host factors that affect CTC viability and apoptosis. Degradation of mRNA is an early event in apoptosis, possibly upstream of caspase activation [35], and therefore might occur in dying cells that are physically intact as judged by lack of DAPI staining. Accumulating evidence suggests that apoptotic CTCs are routinely isolated from cancer patient blood samples. Using the CellSeach platform, FISH, and flow cytometry in conjunction with the M30 antibody which recognizes caspase cleaved CK18, several groups have shown that a significant number of CTCs isolated from metastatic prostate cancer patients are apoptotic [34], [36]–[38]. Detection of apoptotic CTCs is not platform or cancer type specific. Using fiber optic array scanning technology and automated fluorescence imaging, many CTCs isolated from metastatic breast cancer patients are apoptotic morphologically and stain positively in fluorescent TUNEL assays [19], [33].

Despite heterogeneity of CTC RNA quality, we were able to perform single cell transcriptome analysis and confirm that the CTCs were prostatic in origin. Expression of the androgen receptor and target downstream genes such as KLK3 and TMPRSS2 in all but one of the CTCs identifies them as being of prostate origin (Fig. 4A). Furthermore, none of the CTCs, or single LNCAP, PC-3 and T24 cell lines sequenced expressed CD45, a marker for WBCs. Larger studies of isolated CTCs will be necessary to understand the degree of cell-to-cell heterogeneity in gene expression as well as the effects of RNA quality on the fidelity of transcript levels measured by whole transcriptome sequencing in single cells.

Pathway analysis confirmed activation of androgen receptor (AR) signaling pathways, which are known to be central to prostate cancer biology. In addition many genes associated with cell cycle regulation and mitotic spindle are up regulated in the CTCs. While high levels of expression of these transcripts might be expected in malignant cells, it is notable that CTCs showing high levels of expression of spindle-associated transcripts were derived from patients on taxane chemotherapy. Taxanes target the mitotic spindle so it is possible that these transcripts were up-regulated in response to chemotherapy and that this up-regulation could mediate response or resistance to taxane chemotherapy. Several of the transcripts identified in the CTCs have been correlated with aggressive behavior in localized prostate cancer (e.g. PLK-1, TOP2A) so it is interesting to observe these markers in cells from patients with highly advanced prostate cancers [39], [40]. MAGE A1 and CTAG1B show a complex pattern of expression in samples of prostate carcinomas [41]. MAGE C2 is expressed in a small percentage of primary prostate cancers with more frequent expression found in metastatic and castration resistant cancer [42]. Finally, it might be possible to use CTCs to identify therapeutic targets for advanced prostate cancer. For example, CTCs from 2 patients expressed high levels of BIRC5 transcripts. BIRC5 encodes the bi-functional protein survivin which has both anti-apoptotic and mitotic functions in a cell [43]. Survivin has been implicated in castrate resistant prostate cancer and therapeutic antisense RNA to survivin shows effectiveness in treating castrate resistant prostate cancer [44]. In patient 1, SPINK1 was identified in all 3 CTCs analyzed, and accumulating evidence suggests this defines a subtype of prostate cancer that is susceptible to therapies targeting SPINK1 or EGFR (cetuximab) [30].

Previous molecular characterizations of CTCs target a single or a few disease-associated biomarkers. For example, pooled CTCs have been assayed for HER2 in breast cancer using FISH [32], TMPRRSS2-ERG rearrangements in prostate cancer using RT-PCR [8] and EGFR mutations in non-small cell lung cancer [45]. Microarray-based assessments of gene expression have been carried out on pools of prostate, breast and colon cancer CTCs [46] and whole genome amplification coupled with array comparative genomic hybridization has been used to look at copy number variation in small pools of prostate CTCs [21]. With the exception of FISH, no previous technology has allowed assessment of gene expression of single CTCs, and pooling of samples could obscure cell-to-cell variations in expression that are biologically interesting and important. Our demonstration that mRNA-Seq can be carried out on single CTCs, and development of a platform that allows isolation of highly pure individual CTCs offers an opportunity to advance understanding of gene expression in individual CTCs and test whether CTC genomic information can be used in clinical decision making.

## Supporting Information

Table S1 **FPKM values for 23,139 RefSeq RNAs expressed in LNCaP cells and prostate CTCs.** For each cell, the FPKM of RefSeq RNAs were reported.
(XLSX)

Table S2 **FPKM distributions for 23,139 RefSeq RNAs expressed in LNCaP cells and prostate CTCs.** For each cell, the distribution of the FPKM of RefSeq RNAs is calculated.
(XLSX)

Table S3 **CTC gene list.** Manual thresholding was used to identify genes for which the FPKM was at least 100-fold higher in at least 2 of the CTCs compared to normal prostate tissue and that contained at least 10 mapped reads in at least one sample. Gene names are listed alphabetically. CTCs for patients 1,2,3 and 4 are listed across the top of the table.
(XLS)

Table S4 **Clinical history for patient 1, 2, 3, and 4.** Patient age, KLK-3 (PSA) levels and treatment regimens at time of blood collection are listed.
(XLS)
